# Ultrasound Microbubbles Enhance the Neuroprotective Effect of Mouse Nerve Growth Factor on Intraocular Hypertension-Induced Neuroretina Damage in Rabbits

**DOI:** 10.1155/2016/4235923

**Published:** 2016-11-22

**Authors:** Xiaoli Shen, Lina Huang, Dahui Ma, Jun Zhao, Yi Xie, Qiang Li, Aineng Zeng, Kun Zeng, Ruyin Tian, Tianfu Wang, Siping Chen

**Affiliations:** ^1^Affiliated Shenzhen Eye Hospital of Jinan University, Shenzhen, Guangdong, China; ^2^Shenzhen Key Laboratory of Ophthalmology, Shenzhen, Guangdong Province, China; ^3^School of Ophthalmology & Optometry Affiliated to Shenzhen University, Shenzhen, Guangdong, China; ^4^Shenzhen People's Hospital, Shenzhen, Guangdong, China; ^5^Shenzhen University, Shenzhen, Guangdong, China

## Abstract

Ultrasound microbubble combined optic protection drugs have obvious protective effect on optic nerve damage. This way of targeting drug delivery is becoming more simple, not through the whole body metabolism, avoiding drug via blood circulation when facing the decomposition and the environment in the interference and destruction process of drugs, to maximize the guarantee to reach target organs of drug concentration and to reache the maximum therapeutic effect. The technique of ultrasound microbubbles is safe, controllable, nonimmunogenic, and repeatable. It provides us with a novel idea in the administration of neuroprotective drugs.

## 1. Introduction 

The major pathophysiological feature of glaucoma is optic nerve damage, with progressive apoptosis of the retinal ganglion cells (RGCs) being the final pathway [1]. This apoptosis can be blocked; the damaged RGCs can regenerate; and their functions can be restored by neuroprotective molecules. Nerve growth factor (NGF) has the ability to promote development, differentiation, and regeneration of central or peripheral neurons [2–5].

Therapeutic neuroprotective compounds are commonly administered orally, intravenously, or intramuscularly and reach ocular tissue via the systemic circulation. However, blood-eye barrier brings about a low concentration of the drugs in retina and optic nerve, leading to low efficacy and limited application of neuroprotective drugs [6]. Therefore, the development of an efficient drug-delivery system is essential to make neuroprotective drugs more effective.

The recent study in microbubble (ultrasound contrast agent) inspires us to use it as a tool for topical drug-delivery. Microbubbles are blasted using ultrasound with specific energy, so that the drugs can be directly released at the target cells [7, 8]. We hypothesized that combined usage of ultrasound microbubble could increase the protective effect of mNGF against optic nerve damage due to intraocular hypertension. To verify the availability of this administration, we firstly establish an optic nerve damage model due to intraocular hypertension in rabbits and then treat the rabbits with a combination of ultrasound microbubble and mNGF.

## 2. Materials and Methods

### 2.1. Subjects

The animal studies were conducted in compliance with the ARVO statement for the use of animals, and all animal experiments were performed under protocols approved by the Institutional Animal Care of Shenzhen Eye Hospital. Forty healthy New Zealand's rabbits (1.5 kg in body weight) were purchased from Medical Experimental Animal Center of Guangdong Province and maintained in Shenzhen University. Breeding condition was maintained clean and ventilated, with stable temperature and humidity.

### 2.2. Main Reagents and Instruments

**Figure 1:**
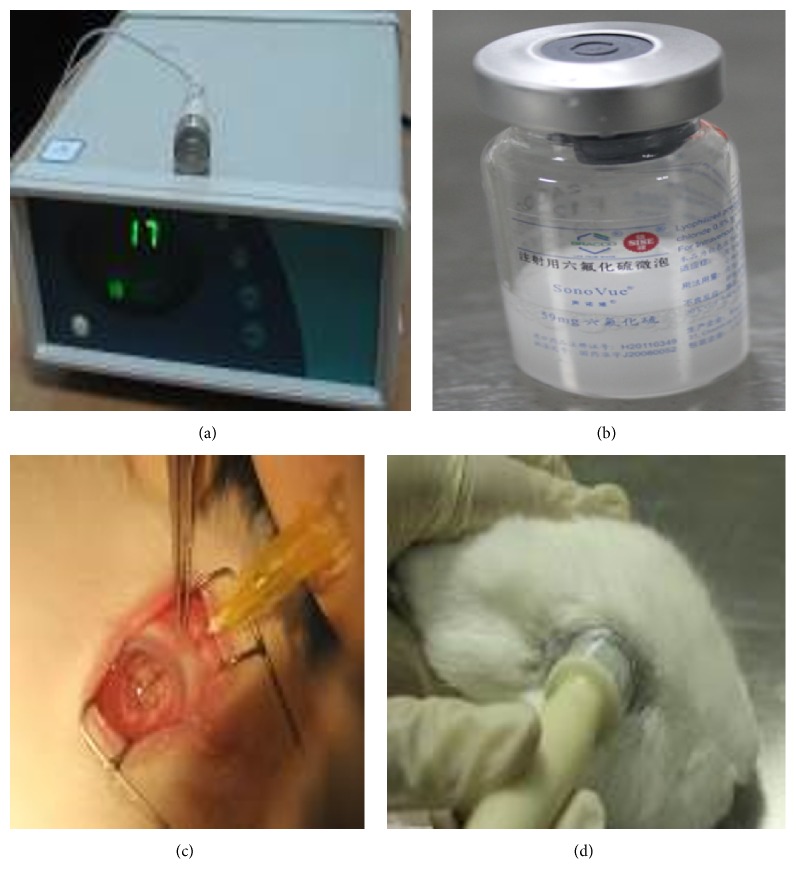
Preparation of ultrasound microbubble. (a) Ultrasonic instrument. (b) SonoVue. (c) mNGF injected into the vitreous. (d) Ultrasound irradiation on the rabbit's eye.

The following was used in experiments: SonoVue (Bracco Co. Ltd., Italy), injection mouse nerve growth factor (Sinobioway Medicine Co. Ltd., China), carbomer-940 (Beijing Guoren Yikang Technology Co. Ltd., China), electrophysiological system (Roland Consult, Germany), optical microscope (Olympus, Japan), transmission electron microscope (FEI, USA), and ultrasonic instrument (Chongqing Medical University, China). The ultrasonic instrument has a center frequency of 1 MHz, with probe diameter of 1 cm. Its intensity ranges from 0 to 3.0 W/cm^2^, with continuous or pulse transmission (Figure 1(a)).

### 2.3. Preparation of the Microbubble Suspension

We slowly injected 5 mL SonoVue into 0.9% saline and shook it to generate foams. Then the microbubble was formed with concentration of 2 × 10^8^/mL, diameter of 2.5 microns, and osmotic pressure of 290 Osm/kg (the same as human plasma) (Figure 1(b)). The microbubble solution should be used within 6 h after preparation.

### 2.4. Preparation of the mNGF Suspension

Mouse nerve growth factors (mNGF) were purchased from Xiamen Beida Biological Engineering Company (Beida, China). A bottle of dried powder of mNGF (18 *μ*g) was mixed with 0.1 mL of 0.9% sodium chloride (18 *μ*g/0.1 mL) before use.

### 2.5. Grouping

A total of 40 New Zealand's rabbits were randomly divided into 5 groups (8 rabbits per group): group A (control group; 0.1 mL microbubble injected into the vitreous), group B (intraocular hypertension control), group C (0.1 mL mNGF injected into the vitreous) (Figure 1(c)), group D (0.1 mL mNGF injected into the vitreous with ultrasound irradiation) (Figure 1(d)), and group E (0.1 mL microbubble and mNGF injected into the vitreous with ultrasound irradiation). Each treatment was given once a week for 3 weeks. Ultrasound irradiation was performed for 60 s at a frequency of 1 MHz and intensity of 0.5 W/cm^2^ [9].

### 2.6. Preparation of Intraocular Hypertension Model

Animals were anesthetized by injection of pentobarbital sodium (3%, 1 mL/kg). 0.2 mL aqueous humor was extracted from anterior chamber; 0.2 mL compound carbomer solution (0.3%) was then transfused in [10]. Record the intraocular pressure (IOP) of the experimental animals. If IOP < 22 mmHg, then the carbomer solution injection was repeated once more. The model should be considered as success if IOP > 22 mmHg, maintained for 4 weeks. Tono-Pen (a pen type tonometer) was used to measure IOP every day.

### 2.7. Flash Visual Evoked Potential Measurements

The animals were anesthetized with 2% pentobarbital sodium (20 mg/kg). Reference electrode was inserted subcutaneously at midpoint between two eyes, recording electrode at midpoint between two ears, grounding electrode behind the right ear. We use Roland electrophysiological system to record flash visual evoked potential (F-VEP). According to ISCEV standard for clinical visual evoked potentials [11], we used white flash stimulation as colorless background with pass-band of 1~300 Hz, using gray flip checkerboard as stimulus signal. The latency and amplitude of *P*_100_ in each animal were recorded every 10 minutes, so in half an hour we obtain three values, and the average of the values was chosen as statistics data.

### 2.8. Histology Examination

After 4 weeks, the rabbits were sacrificed and the retina and optic nerve were collected for hematoxylin and eosin (HE) staining, retinal thickness measurement, retinal ganglion cell (RGC) counting, and transmission electron microscopy (TEM).

### 2.9. RGCs Counting

After 4 weeks, the rabbits were sacrificed and the retina was collected. With 1% toluidine blue stained retina 10~15 min, which was observed under optical microscope. The retina was divided into four quadrants, namely, the supratemporal, the infratemporal, the superior nasal, and the inferior nasal quadrants. Each quadrant was also divided into three parts, namely, the central, the middle, and the surrounding area. In each partition we randomly choose three points to calculate RGC numbers.

### 2.10. Statistical Analysis

The comparison of IOP was conducted by* t*-test, comparison of latency, amplitude of *P*_100_, and retinal thickness and RGC counting was conducted by one-way ANOVA, using SPSS16.0 software. The value of *P* < 0.05 was considered as statistical significance.

## 3. Results

### 3.1. IOP Measurement

IOP of group B at 1, 2, and 4 weeks was significantly higher than that of group A (Table 1).

### 3.2. F-VEP Detection

Compared to group B, the F-VEP showed a statistically significant decrease in latency and increase in amplitude of *P*_100_ in groups C and D, and in group E there was more significant difference (*P* < 0.05) (Table 2).

### 3.3. Retina HE Staining (Figure 2)

*Group A*. Each layer of retina was clear and arranged orderly. From top to bottom it was as follows: retinal ganglion cell layer, inner plexiform layer, inner nuclear layer, the outer plexiform layer, outer nuclear layer, photoreceptor cell layer, and retinal pigment epithelium. Retinal ganglion cells presented single permutation with large, round, or oval nucleus but without vacuolar degeneration. Inner plexiform layer was thick with net-like structure (Muller cells inside). Outer plexiform layer was thinner than inner plexiform layer. In outer nuclear layer, nuclei were dark dyeing, compactly arranged.

*Group B*. Each layer of retina had structural distortion, lacking unity and coherence. Retinal ganglion cells decreased in number, with obvious vacuolar degeneration. Inner plexiform layer and outer plexiform layer became thinner. In inner nuclear layer, nuclei were shallow dyeing, loosely arranged.

*Groups C and D*. In these two groups, we observed similar manifestation in cellular morphology. Each layer of retina was distinct and arranged orderly, relatively. Number of retinal ganglion cells was higher than that in group B. Sporadic vacuolar degeneration could still be seen in retinal ganglion cells. Inner plexiform layer and outer plexiform layer became thinner as well. In inner and outer nuclear layer, nuclei were less dyeing, loosely arranged.

*Group E*. Each layer of retina was clear and arranged orderly. Number of retinal ganglion cells was higher than that in groups C and D. Few vacuolar degeneration could be seen in retinal ganglion cells. The thickness of inner plexiform layer and outer plexiform layer was nearly in normal range. In inner and outer nuclear layer, nuclei were less dyeing, loosely arranged.

### 3.4. Retinal Thickness Measurement and RGCs Counting

Compared to groups C and D, group E had significantly thicker retina and higher retinal RGCs counts (*P* < 0.05) (Table 3).

### 3.5. Ultrastructure of Retina and Optic Nerve

#### 3.5.1. Retinal Ultrastructure (Figure 3)

*Group A*. Structure of photoreceptor cells was clear; rod and cone cells were arranged in alignment. Ganglion cells were round or ovoid, with obvious nuclei. Inside they were full of organelles as mitochondria, rough endoplasmic reticulum, golgi apparatus, and so forth.

*Group B*. Part of photoreceptor cells was rupture, with tumid and vacuolar degenerative mitochondria. Rod outer segments had fuzzy skyline. Ganglion cells decreased in numbers and in microfilament and microtubules components. Organelles such as mitochondria, rough endoplasmic reticulum, or golgi apparatus almost disappeared.

*Groups C and D*. Similar manifestation in cellular morphology was also observed in groups C and D. Arrangement of photoreceptor cells was mildly disordered. There were no vacuolar degenerative mitochondria. Ganglion cells decreased in numbers, but in the nuclei there was homogeneous chromatin. Organelles such as mitochondria, rough endoplasmic reticulum, or golgi apparatus could be seen with mild degeneration.

*Group E*. Structure of photoreceptor cells was distinct; rod and cone cells arranged in alignment, without obvious degeneration. Ganglion cells were nearly normal in structure. Inside there were clear organelles as mitochondria, rough endoplasmic reticulum, golgi apparatus, and so forth.

#### 3.5.2. Optic Nerve Ultrastructure (Figure 4)

*Group A*. The structure of myelin sheath was complete. In the axoplasm, microtubules, microfilaments, and organelles such as mitochondria could be seen explicitly.

*Group B*. The dissolved myelin sheath was loose. In the axoplasm, microtubules and microfilaments became swallowing. Vacuolar degenerative mitochondria could be seen.

*Groups C and D*. Part of myelin sheath was attenuation. In the axoplasm, microtubules and microfilaments became mildly swallowing. Vacuolar degenerative mitochondria could also be seen.

*Group E*. The structure of myelin sheath was complete but fair-arranged. In the axoplasm, microtubules, microfilaments, and organelles such as mitochondria could be seen without degeneration.

## 4. Discussion

Microbubble as new drug carriers can produce a variety of biological effects after ultrasonic irradiation. Its cavitation and sonoporation effect mean that it can generate reversible holes on cell membranes. Therefore drugs can easily enter into cells so as to increase the permeability [12]. The technique of ultrasound microbubbles is safe, controllable, nonimmunogenic, repeatable, and well targeted. It provides us with a novel idea in the administration of neuroprotective drugs. However, ultrasound microbubble can also generate certain harmful biological results, including fracture of tissue, bleeding, intravascular hemolysis, and even cell death, which relates to its cavitation effect [13]. Recognizing that a high ultrasonic energy or long irradiation time might cause tissue damage, we have optimized suitable ultrasound parameters for rabbits in pilot study, that is, the frequency of 1 MHz, intensity of 0.5 W/cm^2^, and duration of 60 s [9].

We choose anterior chamber injection of carbomer solution to establish high intraocular pressure model. IOP of carbomer group at 1, 2, and 4 weeks was significantly higher than that of normal group. Thus animal model of glaucoma was successfully established. We observed function and structure of the rabbits' retina and optic nerve. Visual evoked potential (VEP) is a sensitive method for evaluating nerve damage, primarily reflecting lesions between retina and visual cortex. The latency and amplitude of flash visual evoked potential (F-VEP) mainly reflect the function of optic nerve myelin and axons [14]. Our results showed that, due to intraocular hypertension, there was an increase in latency and decrease in amplitude of *P*_100_, meaning an impairment of signal transduction [15]. After intravitreal injection of mNGF, the latency decreased, accompanied with amplitude increase. There was no significant difference in latency and amplitude of *P*_100_ between mNGF+ultrasonic irradiation group and mNGF group (*P* > 0.05), which indicates that ultrasonic irradiation alone does not enhance effect of mNGF. Compared to mNGF treatment only, a more significant decrease in latency and increase in amplitude of *P*_100_ were seen in mNGF+ultrasound microbubbles group. It proved the importance of cavitation effect made by microbubbles. In the test of retinal thickness and RGCs counting, similar results could be seen in our experiments. Compared to mNGF treatment only, the treatment with addition of ultrasound microbubbles gave rise to less retina damage.

Light microscope observation showed that intraocular hypertension could lead to disordered phenomenon and cellular vacuolar degeneration of retina. Electron microscope observation provided us with a more visual result; it revealed that intraocular hypertension could lead to various degrees of cell edema, RGCs loss, optic nerve myelin sheath damage, and decreases in mitochondria, microtubules, and microfilaments. With treatment of mNGF, there was less tissue damage. Adding ultrasound irradiation cannot increase the protective effect, but, adding microbubbles also, tissue damage distinctly decreased.

Recently, ultrasound microbubbles have achieved great progress in the experimental study of genes or drugs carriers [16–20]. The findings of our study are consistent with previous research. Li et al. [16] investigated the expression levels of green fluorescence protein (GFP) into retinal ganglion cells (RGCs) in vitro by ultrasound-mediated microbubble destruction (UMMD) and assess the effect of bcl-xl gene on N-methyl-D-aspartate- (NMDA-) induced apoptosis in the cultured RGCs by UMMD. Their results showed that ultrasound combined with microbubbles enhanced gene transfection to the cultured cells in some conditions. The average transfection rate of pEGFP-N1 with UMMD was 25%. Both ultrasound and microbubble had no effect on cell viability. The expression of bcl-xl protein in transfected and nontransfected RGCs was significantly different. Less apoptotic bodies and no representative DNA fragment were detected in the treatment group. Xie et al. [17] reported that UMMD can effectively and safely enhance recombinant adenoassociated virus delivery to RGCs in rats, and it may serve as a novel gene delivery method in gene therapy for glaucomatous optic neuroprotection. Fu et al. [18] investigated the protective effect of ultrasound microbubbles mediated transfection of brain-derived neurotrophic factor (BDNF) into the retina and visual cortex on RGCs after optic nerve injury in rats. They found that survival rate of RGCs was higher in the study group that underwent ultrasound microbubble-mediated transfection of BDNF. Liu et al. [19] investigated the effects of ciliary neurotrophic factor (CNTF) gene mediated by ultrasound microbubbles intraocular transfer on visual function and RGCs after optic nerve injury. Their results showed that the latency of P1 was significantly shorter in the ultrasound microbubble group compared with the plasmid group and plasmid combined with ultrasound group; and the amplitude of P1 was significantly increased in the ultrasound microbubbles group. They also found that the average counts of RGCs and the expression level of CNTF mRNA were significantly higher in the ultrasound microbubble group than in the plasmid group and plasmid combined with ultrasound group. Their findings indicated that ultrasound microbubbles can enhance the transfection and expression of the CNTF gene in the eye, protect against early damage of RGCs in rats, and effectively promote the recovery of visual function. Yang et al. [20] evaluated the protection effects of ultrasonic microbubbles combined with memantine on rat RGCs after optic nerve injury and found that the RGC count was significantly higher in the group treated with ultrasound microbubbles combined with intravitreal injection of memantine, indicating that the protective effect of ultrasound microbubbles combined with memantine was greater compared to intravitreal injection of memantine alone.

In conclusion, mNGF can decrease optic nerve damage due to intraocular hypertension. Combined usage of microbubble with ultrasound irradiation can strengthen its protective effect.

## Figures and Tables

**Figure 2:**
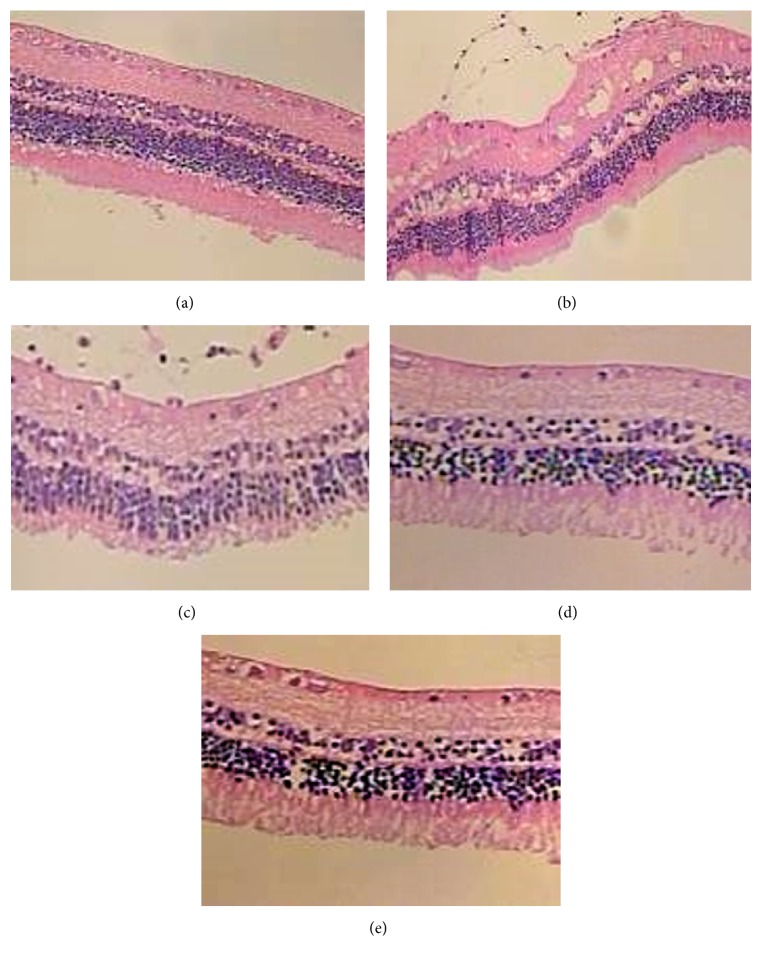
Histopathological structure of the retina. (a) Retinal ganglion cells presented single permutation with large, round, or oval nucleus but without vacuolar degeneration. Inner plexiform layer was thick with net-like structure (Muller cells inside). Outer plexiform layer was thinner than inner plexiform layer. In outer nuclear layer, nuclei were dark dyeing, compactly arranged. (b) Each layer of retina had structural distortion, lacking unity and coherence. Retinal ganglion cells decreased in number, with obvious vacuolar degeneration. Inner plexiform layer and outer plexiform layer became thinner. In inner nuclear layer, nuclei were shallow dyeing, loosely arranged. ((c), (d)) Each layer of retina was distinct and arranged orderly, relatively. Number of retinal ganglion cells was higher than that in group B. Sporadic vacuolar degeneration could still be seen in retinal ganglion cells. Inner plexiform layer and outer plexiform layer became thinner as well. In inner and outer nuclear layer, nuclei were less dyeing, loosely arranged. (e) Each layer of retina was clear and arranged orderly. Number of retinal ganglion cells was higher than that in groups C and D. Little vacuolar degeneration could be seen in retinal ganglion cells. The thickness of inner plexiform layer and outer plexiform layer was nearly in normal range. In inner and outer nuclear layer, nuclei were less dyeing, loosely arranged.

**Figure 3:**
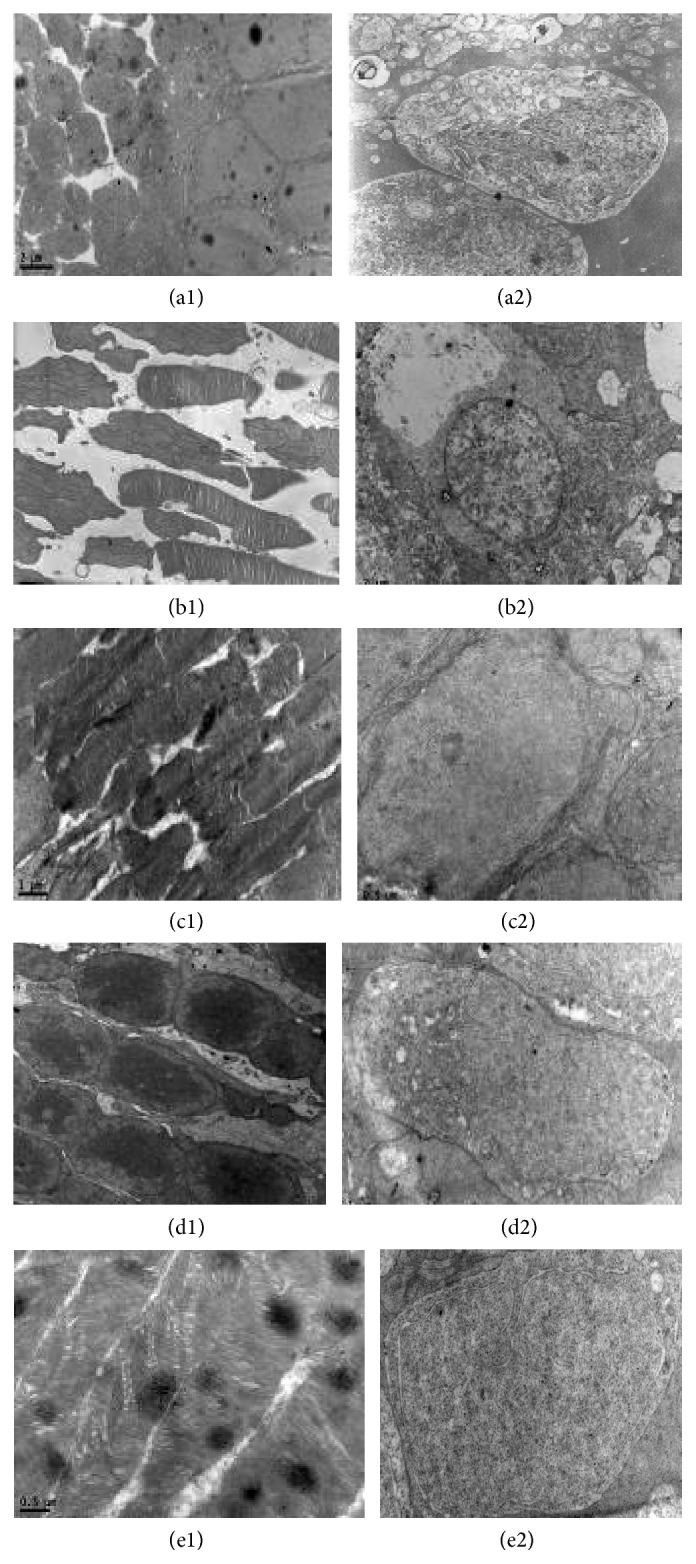
Ultrastructure of retina. (a1) Structure of photoreceptor cells was clear; rod and cone cells were arranged in alignment. (a2) Ganglion cells were round or ovoid, with obvious nuclei. Inside they were full of organelles as mitochondria, rough endoplasmic reticulum, golgi apparatus, and so forth. (b1) Part of photoreceptor cells was rupture, with tumid and vacuolar degenerative mitochondria. Rod outer segments had fuzzy skyline. (b2) Ganglion cells decreased in numbers and in microfilament and microtubules components. Organelles such as mitochondria, rough endoplasmic reticulum, or golgi apparatus almost disappeared.** (**(c1), (d1)) Arrangement of photoreceptor cells was mildly disordered. There were no vacuolar degenerative mitochondria. ((c2), (d2)) Ganglion cells decreased in numbers, but in the nuclei there was homogeneous chromatin. Organelles such as mitochondria, rough endoplasmic reticulum, or golgi apparatus could be seen with mild degeneration. (e1) Structure of photoreceptor cells was distinct; rod and cone cells arranged in alignment, without obvious degeneration. (e2) Ganglion cells were nearly normal in structure. Inside there were clear organelles as mitochondria, rough endoplasmic reticulum, golgi apparatus, and so forth.

**Figure 4:**
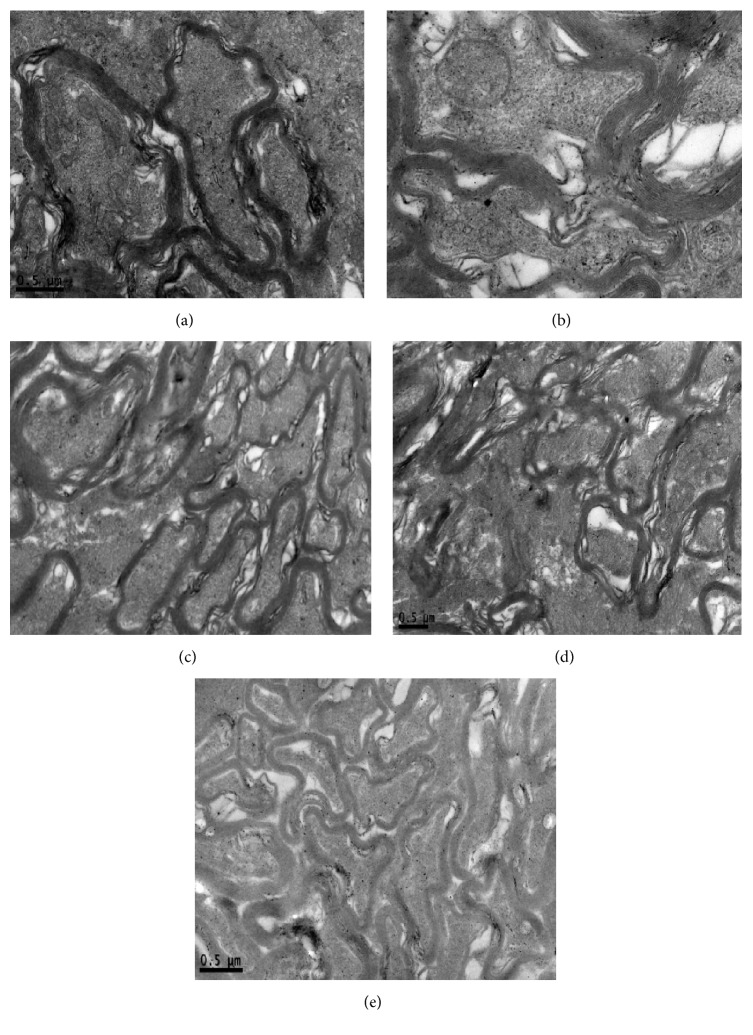
Ultrastructure of optic nerve. (a) The structure of myelin sheath was complete. In the axoplasm, microtubules, microfilaments, and organelles such as mitochondria could be seen explicitly. (b) The dissolved myelin sheath was loose. In the axoplasm, microtubules and microfilaments became swallowing. Vacuolar degenerative mitochondria could be seen. (c) (d) Part of myelin sheath was attenuation. In the axoplasm, microtubules and microfilaments became mildly swallowing. Vacuolar degenerative mitochondria could also be seen. (e) The structure of myelin sheath was complete but fair-arranged. In the axoplasm, microtubules, microfilaments, and organelles such as mitochondria could be seen without degeneration.

**Table 1:** Comparison of IOP between group A and group B (mmHg).

Time points	Before treatment	1 week	2 weeks	4 weeks
Group A	13.6 ± 1.5	13.6 ± 1.8	13.4 ± 1.7	13.3 ± 1.4
Group B	15.0 ± 2.0	33.4 ± 2.8	34.1 ± 2.5	34.8 ± 2.2
*t*	−1.561	−17.845	−21.308	−22.71
*P*	0.137	0.000	0.000	0.000

**Table 2:** Comparison of latency and amplitude of *P*_100_.

	Latency (ms)	Amplitude (nV)
Group A	46.20 ± 6.90	15.90 ± 2.48
Group B	125.00 ± 18.70	5.50 ± 3.03
Group C	102.10 ± 18.77^*∗*^	9.30 ± 3.13^*∗*^
Group D	102.50 ± 17.87^*∗*^	9.20 ± 3.42^*∗*^
Group E	63.80 ± 8.35	11.37 ± 2.84

^*∗*^In one-way ANOVA, there was no statistically significant difference about the mean value of latency and amplitude between groups C and D (*P* > 0.05).

**Table 3:** Retinal thickness and RGCs counting.

	Retinal thickness (*μ*m)	Number of RGCs
Group A	289.30 ± 2.39	26.04 ± 0.70
Group B	239.15 ± 2.68	14.97 ± 1.30
Group C	254.50 ± 3.03^*∗*^	19.33 ± 0.78^*∗*^
Group D	257.05 ± 2.28^*∗*^	20.25 ± 0.98^*∗*^
Group E	269.50 ± 3.00	23.97 ± 0.90

^*∗*^In one-way ANOVA, there was no statistically significant difference about the mean value of retinal thickness and number of RGCs between groups C and D (*P* > 0.05).
